# The Gαh-PLCδ1 signaling axis drives metastatic progression in triple-negative breast cancer

**DOI:** 10.1186/s13045-017-0481-4

**Published:** 2017-06-02

**Authors:** Shang-Pen Huang, Pei-Yao Liu, Chih-Jung Kuo, Chi-Long Chen, Wei-Jiunn Lee, Yu-Hui Tsai, Yuan-Feng Lin

**Affiliations:** 1 Graduate Institute of Clinical Medicine, College of Medicine, Taipei Medical University, 250 Wu-Hsing Street, 110 Taipei, Taiwan; 2 Department of Veterinary Medicine, National Chung Hsing University, Taichung, Taiwan; 3 Department of Pathology, Wan Fang Hospital, Taipei Medical University, Taipei, Taiwan; 4 Department of Urology, School of Medicine, Taipei Medical University, Taipei, Taiwan; 5 Department of Medical Education and Research, Wan Fang Hospital, Taipei Medical University, Taipei, Taiwan; 6 Graduate Institute of Medical Sciences, College of Medicine, Taipei Medical University, Taipei, Taiwan

**Keywords:** Gαh, Metastasis, PLCδ1, Protein-protein interaction, Triple-negative breast cancer

## Abstract

**Background:**

Distant metastasis of triple-negative breast cancer (TNBC) to other organs, e.g., the lungs, has been correlated with poor survival rates among breast cancer patients. Therefore, the identification of useful therapeutic targets to prevent metastasis or even inhibit tumor growth of TNBC is urgently needed. Gαh is a novel GTP-binding protein and known as an inactive form of calcium-dependent tissue transglutaminase. However, the functional consequences of transamidating and G-protein activities of tissue transglutaminase in promoting cancer metastasis are still controversial.

**Methods:**

Kaplan-Meier analyses were performed to estimate the prognostic values of Gαh and PLCδ1 by utilizing public databases and performing immunohistochemical staining experiments. Cell-based invasion assays and in vivo lung colony-forming and orthotropic lung metastasis models were established to evaluate the effectiveness of interrupting the protein-protein interaction (PPI) between Gαh and PLCδ1 in inhibiting the invasive ability and metastatic potential of TNBC cells.

**Results:**

Here, we showed that the increased level of cytosolic, not extracellular, Gαh is a poor prognostic marker in breast cancer patients and correlates with the metastatic evolution of TNBC cells. Moreover, clinicopathological analyses revealed that the combined signature of high Gαh/PLCδ1 levels indicates worse prognosis in patients with breast cancer and correlates with lymph node metastasis of ER-negative breast cancer. Blocking the PPI of the Gαh/PLCδ1 complex by synthetically myristoylated PLCδ1 peptide corresponding to the Gαh-binding interface appeared to significantly suppress cellular invasiveness in vitro and inhibit lung metastatic colonies of TNBC cells in vivo.

**Conclusions:**

This study establishes Gαh/PLCδ1 as a poor prognostic factor for patients with estrogen receptor-negative breast cancers, including TNBCs, and provides therapeutic value by targeting the PPI of the Gαh/PLCδ1 complex to combat the metastatic progression of TNBCs.

**Electronic supplementary material:**

The online version of this article (doi:10.1186/s13045-017-0481-4) contains supplementary material, which is available to authorized users.

## Background

Triple-negative breast cancers (TNBCs) are a diverse and heterogeneous group of tumors that lack estrogen and progesterone receptors and HER2 gene amplification. Because they do not express these receptors, hormonal (e.g., tamoxifen) and targeted (e.g., Herceptin) therapies are not effective in treating TNBCs. Moreover, the majority of TNBCs are highly malignant, and only a subgroup responds to conventional chemotherapy with favorable prognosis [1–3]. Therefore, drug resistance and metastatic progression are major clinical issues in the successful treatment of TNBCs. Although high-throughput molecular analyses, including sequencing, pathway analyses, and integrated analyses of alterations at the genomic and transcriptomic levels, have improved our understanding of the molecular alterations involved in TNBC development and progression, the proper use of this knowledge for the rational selection of therapy remains challenging.

Gαh is a novel GTP-binding protein that is an inactive form of calcium-dependent tissue transglutaminase; a multifunctional protein that is ubiquitously expressed in various tissues [4]. Gαh transmits signals from activated G protein-coupled receptors, e.g., α1-adrenoceptor [5] and follicle-stimulating hormone receptor [6], to phospholipase C-δ1 (PLC-δ1) via protein-protein interactions (PPIs). Activation of the Gαh/PLC-δ1 pathway has been shown to elevate the concentration of intracellular inositol 1,4,5-triphosphate (IP_3_) and calcium [7], both of which are important secondary messengers in signal transduction and promote cell survival, growth, and invasion [8, 9]. In contrast, an abnormal increase in intracellular calcium due to cellular damage or other stressors has been shown to activate the transglutaminase feature of Gαh activity to catalyze the crosslinking of cellular proteins and ultimately induce cell death [10]. Recently, Gαh overexpression has been observed in breast [11, 12], colon [13], and ovarian cancers [14]; non-small cell lung cancer [15]; and renal cell carcinoma [16] and has been shown to be associated with advanced disease stages and metastatic spread. Importantly, the loss of the GTP-binding, but not transamidating, activity of Gαh was shown to inhibit cancer metastasis in malignant tumor cells [17], implying the critical importance of Gαh/PLC-δ1-dependent signaling in cancer progression. These findings prompted us to evaluate the feasibility of therapeutic inhibition of the PPI of Gαh with PLC-δ1 in combating cancer metastasis.

Here, we find that Gαh expression is causally associated with the metastatic potential of TNBC cells *in vitro* and *in vivo* and is strongly correlated with poor distant metastasis-free survival probability in patients with breast cancer. Notably, loss of the G protein function, but not the transglutaminase activity, abolished the Gαh-induced metastatic progression of TNBC cells. Interestingly, blocking the PPI between Gαh and PLC-δ1 using a synthetic peptide blocker corresponding to the binding-interface of Gαh effectively inhibited the metastatic progression of TNBC cells in vitro and in vivo. These findings provide a potential new strategy to develop anti-cancer agents for overcoming cancer metastasis.

## Methods

### Cell lines and cell culture condition

TNBC cell lines MDA-MB-468, MDA-MB-231, and MDA-MB-436 were cultured in Leibovitz’s (L-15) medium (Gibco Life Technologies, Grand Island, NY, USA) supplemented with 10% fetal bovine serum (FBS, Invitrogen) and incubated at 37 °C with free gas exchange with atmospheric air. TNBC cell lines HCC1143, HCC1599, HCC70, and HCC38 were cultured in RPMI-1640 medium (Gibco Life Technologies) with 10% FBS and incubated at 37 °C with 5% CO_2_. HS578T and 293T cells were cultured in DMEM with 10% FBS and incubated at 37 °C with 5% CO_2_. All cell lines were obtained from American Type Culture Collection (ATCC). All cells were routinely authenticated on the basis of short tandem repeat (STR) analysis, morphologic, and growth characteristics and mycoplasma detection.

### Public database

Raw data for pathological information and prognostic values of the Gαh gene were downloaded from the PrognoScan database (http://www.prognoscan.org/). Datasets with *p* value < 0.05 by Cox regression analysis were included in a meta-analysis for Gαh gene expression. Transcriptional profiling for Gαh was obtained from The Cancer Genome Atlas (TCGA) database and subjected to statistical analysis against differential levels of Gαh mRNA in the subgroups. Kaplan-Meier analysis and the differential analysis of Gαh gene expression were performed on the SurvExpress website.

### In vitro invasion assay

For invasion assays, Boyden chambers (Neuro Probe, Gaithersburg, MD, USA) were used according to the manufacturer’s protocol. Briefly, a polycarbonate membrane (Neuro Probe) was pre-coated with 10 μg of human fibronectin (Sigma-Aldrich, St Louis, MO, USA) on the lower side and Matrigel on the upper side. Cells (1.5 × 10^4^) were seeded in the top chamber in 50 μl of low-serum medium (0.1% FBS) containing drugs or DMSO. After 16 h, stationary cells were removed from the top surface of the membrane, whereas the migrated cells on the bottom surface of the membrane were fixed in 100% methanol and stained with 10% Giemsa solution (Merck Biosciences, Mendota Heights, MN, USA) for 1 h. The invaded cells were counted under a light microscope (×400, ten random fields from each well). All experiments were performed in triplicate.

### Western blot analysis

Proteins (100 μg) were boiled for 5 min in SDS sample buffer (62.5 mM Tris [pH 6.7], 1.25% SDS, 12.5% glycerol, and 2.5% β-mercaptoethanol) and separated on 10% SDS-PAGE gels. After transferring to PVDF membranes, the proteins were incubated with antibodies against Gαh (GeneTex, Hsin-Chu, Taiwan), PLC-δ1 (GeneTex), or GAPDH (AbFrontier, San Diego, CA, USA). The immunoreactive bands were visualized using an enhanced chemiluminescence system (GE Healthcare, Pittsburgh, PA, USA).

### GTP-binding assay

Membrane proteins (100 μg) were diluted with 500 μl of 5-fold diluted buffer B (prepared as instructed by the manufacturer of the membrane extraction kit) containing 1 mM sodium orthovanadate, 1 mM dithiothreitol (DTT), and 4% protease inhibitor cocktail (Merck Biosciences). The membrane proteins were then absorbed into GTP-agarose (Sigma-Aldrich) overnight at 4 °C. The GTP-agarose-absorbed complexes/proteins were subsequently analyzed by SDS-PAGE/Western blotting using antibodies against PLC-δ1 or Gαh.

### In situ tTG activity assay

A 50 μl aliquot of cell homogenate proteins (10 μg) in coating buffer (50 mM Tris–HCl [pH 7.4], 150 mM NaCl, 5 mM EDTA, and 5 mM EGTA) was added to each well of a 96-well ELISA plate (Apogent, Portsmouth, NH, USA) and was incubated at 4 °C overnight. To block the coated wells, a 200 μl aliquot of 5% BSA, 0.01% Tween 20, 0.01% SDS in borate-buffered saline (BBS) was added. After an additional 2 h of incubation at 37 °C, the plate was rinsed once with 1% BSA and 0.01% Tween-20 in BBS. To each well, 100 μl of HRP-conjugated streptavidin (Southern Biotech, Birmingham, AL, USA) (1:1000) in 1% BSA and 0.01% Tween-20 in BBS was added, followed by incubation for another 1 h at room temperature (RT). After the wells were rinsed four times with 1% BSA and 0.01% Tween-20 in BBS, the specimen in each well was incubated with 200 μl of TMB substrate (Sigma-Aldrich) for 10–20 min at RT. The reaction was stopped with 50 μl of 3 N HCl, and the absorbance at 450 nm was measured using a microplate spectrophotometer.

### Peptide synthesis

TIPWNSLKQGYRHVHLL, a peptide corresponding to the PLC-δ1 amino acid sequence from 720 to 736, was synthesized with or without myristoylation at the N-terminus and purified to approximately 95% (Mission Biotech, Taipei, Taiwan). Ten millimolar synthetic peptide was dissolved in 1 part of 0.1% trifluoroacetic acid in dd-H_2_O mixed with 1 part 0.1% trifluoroacetic acid in acetonitrile, aliquoted, and kept at −70 °C as a stock solution.

### Plasmid construction

The genes encoding Gαh and PLC-δ1 were amplified from human cDNA (Invitrogen) using standard polymerase chain reaction (PCR) with paired primers (Additional file 1: Table S1) and sub-cloned into the pLAS3w/Ppuro, pLAS3w/Pbsd, or pIRES2-EGFP vector. The Gαh-containing pIRES2-EGFP plasmid was used as a DNA template for site-directed and deletion mutagenesis. PCR for site-directed mutagenesis was performed with paired primers (Additional file 1: Table S1) using a *pfu* polymerase kit (Stratagene, La Jolla, CA, USA) (for R580A) or primer extension method [18] (for W241A). The PCR products were treated with Dpn1 endonuclease (New England BioLabs, Hitchin, Hertfordshire, UK) to digest the methylated parental DNA template. For the construction of a C-terminal deletion mutant of Gαh (∆657-687), a Gαh-containing pIRES2-EGFP plasmid was used as the template to amplify the target sequence using paired primers (Additional file 1: Table S1). The two PCR products were subsequently digested with NheI and EcoRI and then ligated into pLAS3w/Pbsd. The identities of individual clones were verified via double-strand plasmid sequencing.

### Preparation and infection of lentiviral particles

All lentiviral vectors, including pLAS3w/Ppuro and pLAS3w/Pbsd and derivatives of shRNA vector (obtained from the National RNAi Core Facility Platform in Taiwan), were transfected into the packaging cell line 293T along with pMD.G and pCMVΔR8.91 plasmids using a calcium phosphate transfection kit (Invitrogen). After incubation for 48 h, the viral supernatants were collected and transferred to target cells, and the infected cells were cultured in the presence of puromycin and blasticidin (Calbiochem, San Diego, CA, USA) at 5–10 μg/ml in order to select the stably transfected cells.

### Animal experiments

NOD/SCID mice were obtained from the National Laboratory Animal Center in Taiwan and maintained in compliance with the institutional policy. All animal procedures were approved by the Institutional Animal Care and Use Committee at Taipei Medical University.

For the in vivo lung metastatic colonization assay, cells were implanted into mice through tail vein injection at a concentration of 1 × 10^6^ cells/100 μl of PBS. The mice were sacrificed, and the lungs were dissected for histological analyses at the endpoint. The metastatic lung nodules were quantified after H&E staining using a dissecting microscope.

For orthotopic lung metastasis of breast cancer, GFP/luciferase-expressing MDA-MB-231 cells (5 × 10^5^), which were established by virally transducing cells with an EF1 promoter-driven firefly luciferase vector and an IRES-driven EGFP vector, were suspended in 50 μL of PBS and then were subcutaneously inoculated into the abdominal fat pad of each mouse. In vivo tumor images were captured with an IVIS imaging system (Caliper Life Sciences, Alameda, CA, USA) to measure the signal intensity from the GFP/luciferase vector. The mice were humanely sacrificed at the end of the experiments, and lungs were obtained for histological analyses. The tumors at the primary sites were removed for weight measurement.

### Clinical samples and ethics statement

The breast cancer tissues used in this study were from Wan Fang Hospital managed by Taipei Medical University (TMU). Patient information, including gender, age, and histopathological diagnoses, was collected. The surgical specimens had been fixed in formalin and embedded in paraffin before being archived. We used the archived specimens for IHC staining. Follow-up of patients was carried out for up to 60 months. A four-point staining intensity scoring system was devised to determine the relative expression of Gαh and PLC-δ1 in the cancer specimens; the staining intensity score ranged from 0 (no expression) to 3 (maximal expression). All of the IHC staining results were reviewed and scored independently by two pathologists. The study was carried out with the approval of the Institutional Review Boards and with permission from the ethics committees of the institutions involved (TMU-IRB 99049).

### Immunohistochemical staining analysis

Paraffin-embedded tumor sections (3 μm thick) were heated and deparaffinized using xylene and then were rehydrated in a graded series of ethanol with a final wash in tap water. Antigen retrieval was performed with a target retrieval solution (DAKO, Woodbridge, VA, USA) in a decloaking chamber (Biocare Medical, Concord, CA, USA). Endogenous peroxidase activity was quenched by hydrogen peroxide. The sections were then incubated with anti-Gαh and anti-PLC-δ1 antibodies (GeneTex) at 4 °C overnight. The Vectastain ABC peroxidase system (Vector Laboratories, Burlingame, CA, USA) was used to detect the reaction products.

### Statistical analysis

SPSS 17.0 software (Informer Technologies, Roseau, Dominica) was used to analyze statistical significance. Paired *t* test was utilized to compare Gαh gene expression in the cancer tissues and corresponding normal tissues. Pearson’s test was performed to estimate the association between mRNA and protein expression of Gαh and PLC-δ1 by RNA-sequencing and IHC, respectively. Evaluation of survival probabilities were determined by Kaplan-Meier analysis and log-rank test. One-way ANOVA with Tukey’s test was used to estimate the difference in lung colonies and tumor weights in the animal experiments. The Mann-Whitney *U* test was used to analyze the non-parametric data. *p* values < 0.05 in all analyses were considered statistically significant.

## Results

### Gαh upregulation predicts a poor probability of distant metastasis-free survival in breast cancer patients

**Fig. 1:**
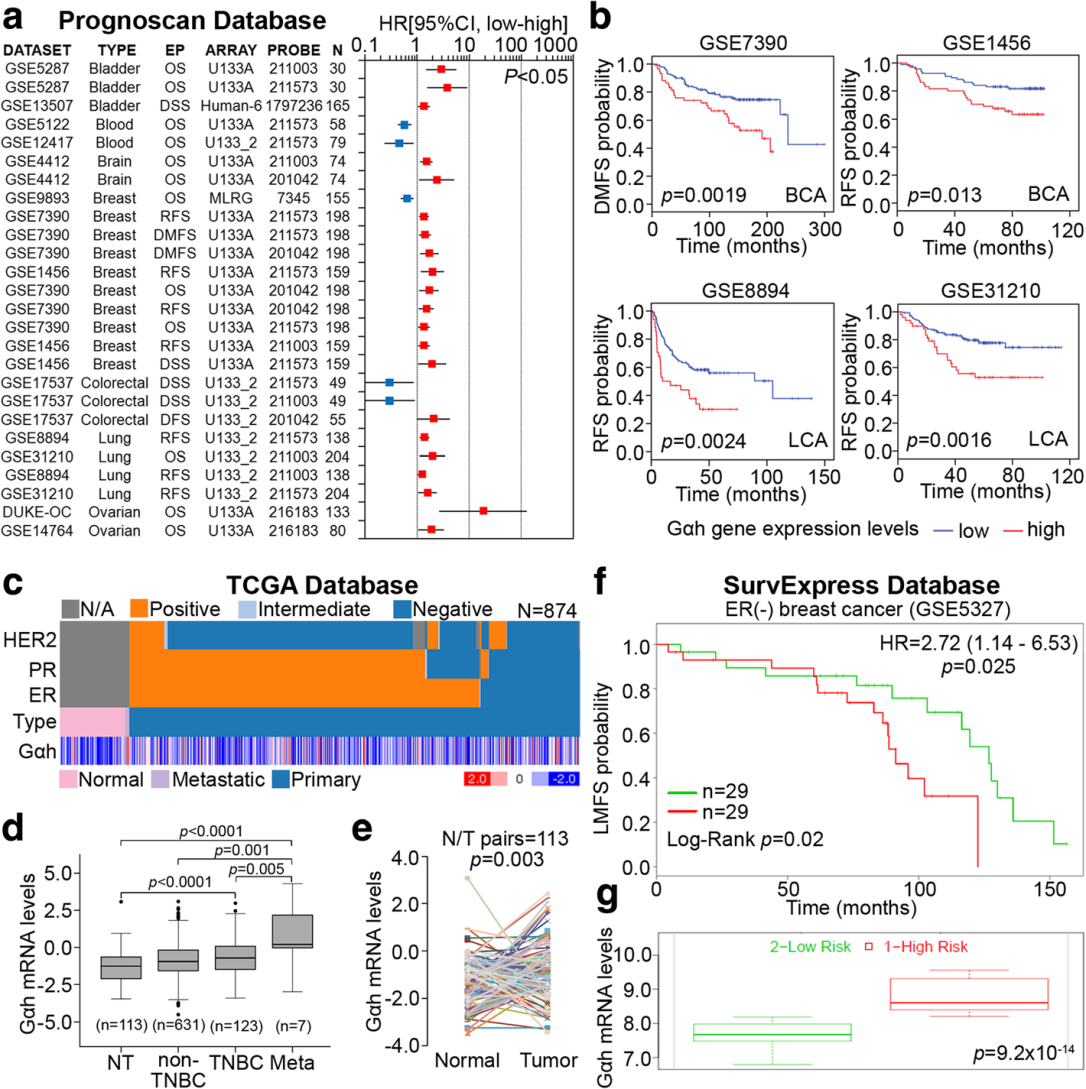
Gαh-OE correlates with the metastatic progression of breast cancer. **a** A global meta-analysis of the expression of Gαh gene (*TGM2*) in clinical cohorts with different cancer types using the PrognoScan database. *EP* denotes endpoint, *N* indicates patient number, and *P* represents the statistical significance by Cox regression. **b** Kaplan-Meier plot for Gαh gene expression from *GSE7390*, *GSE1456*, *GSE8894*, and *GSE31210* datasets in the PrognoScan database to evaluate the probability of distant metastasis-free survival (*DMSF*) or recurrence-free survival (*RFS*). **c** Heatmap of Gαh gene expression in normal tissues and metastatic and primary tumor tissues derived from clinical breast cancer patients in TCGA database. **d** Gαh gene expression in normal tissues and tumor tissues derived from patients with non-TNBC, TNBC, and metastatic breast cancer in TCGA database. **e** Gαh gene expression in paired normal and tumor tissues derived from breast cancer patients in TCGA database. **f** and **g** Kaplan-Meier analysis of Gαh gene levels (**f**) and Gαh gene transcriptional activity (**g**) in cohorts with low and high risk of lung metastasis in estrogen receptor (ER)-negative breast cancer patients from the GSE5327 dataset in the SurvExpress database

To evaluate the clinical relevance of Gαh overexpression (OE), we performed a global prognostic meta-analysis for the Gαh gene (*TGM2*) on microarray data from clinical cohorts with different cancer types using PrognoScan [19]. The data demonstrated that the upregulation of Gαh correlated with poor prognosis as judged by an increased hazard ratio in patients with bladder, brain, breast, lung, or ovarian cancers but not in patients with blood or colorectal cancer (Fig. 1a). Although the lower expression of Gαh transcripts referred to a poor outcome in a few studies, the clinical cohorts with highly invasive breast and lung cancers that expressed an increased level of Gαh transcripts accounted for the reduction in the probabilities of distant metastasis or recurrence-free survival (Fig. 1b). Based on these findings, this study was focused on investigating the oncogenic effect of Gαh overexpression (OE) on promoting the metastatic progression of breast cancer.

Next, we examined the transcriptional profiles of Gαh in clinical tissues from breast cancer patients using The Cancer Genome Atlas (TCGA) database [20]. The data showed that the transcriptional activity of Gαh in TNBC and metastatic breast cancer was higher than that of the non-tumor and non-TNBC tissues (Fig. 1c, d). Moreover, the transcriptional activity of Gαh appeared to be enhanced in cancer tissues compared to the adjacent normal tissues derived from breast cancer patients (Fig. 1e).

To validate the clinical relevance of Gαh expression in the metastatic evolution of TNBCs, we evaluated the prognostic significance of Gαh-OE in the lung metastasis of an estrogen receptor (ER)-negative breast cancer cohort (GSE5327) from the SurvExpress database. The data showed that Gαh-OE indicated a poor probability of lung metastasis-free survival (Fig. 1f) and was notable in breast cancer tissues derived from patients with a higher risk of lung metastasis (Fig. 1g).

### Gαh overexpression promotes the metastatic potential of TNBC cells

**Fig. 2:**
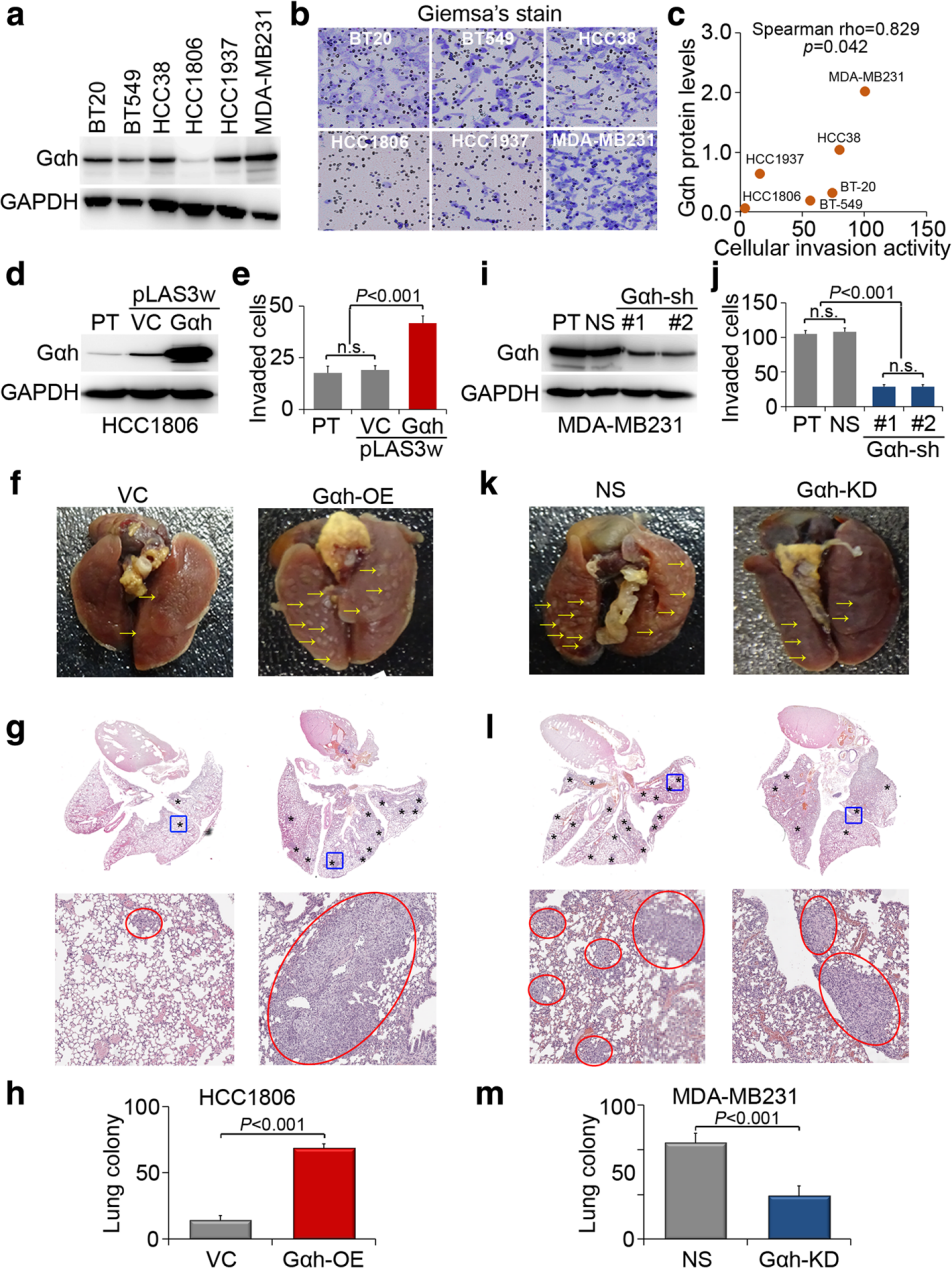
The upregulation of Gαh promotes the metastatic potential of TNBC cells in vitro and in vivo. **a** Western blot analysis for the endogenous expression of Gαh and GAPDH in a panel of TNBC cell lines. **b** Giemsa staining for the invaded cells after 16 h of incubation in a panel of TNBC cell lines. **c** Correlation between endogenous protein levels of Gαh and cellular invasion ability in TNBC cell lines. Statistical significance was analyzed by Spearman’s correlation test. **d** Western blot analysis for the expression of Gαh and GAPDH in parental (*PT*) HCC1806 cells or HCC1806 cells without (vector control, *VC*), or with Gαh overexpression. **e** The invaded cells after 16 h of incubation of the HCC1806 cell variants shown in **d. f**–**h** Lung colony formation assay of HCC1806 cells without (*VC*) or with Gαh overexpression (*Gαh-OE*). Lungs were obtained from mice (*n* = 5) after 4 weeks of tail vein injection (**f**) and then were subjected to H&E staining (**g**). **h** Tumor nodules in the representative images indicated by *arrows* in **f** were counted. **i** Western blot analysis for the expression of Gαh and GAPDH in parental (*PT*) MDA-MB-231 cells or MDA-MB-231 cells transfected with non-silencing (**NS**) or 2 independent Gαh-targeting shRNAs. In **d** and **i**, GAPDH was used as the internal control for protein loading. **j** The invaded cells after 16 h of incubation from the MDA-MB-231 cell variants shown in **i. k**–**m** Lung colony formation assay of MDA-MB-231 cells transfected with NS or Gαh-targeting shRNAs. **k** Lungs were obtained from mice (*n* = 5) 4 weeks after tail vein injection **l** and then subjected to H&E staining. **m** Tumor nodules in the representative images indicated by arrows in E were counted

To investigate the association between the expression of Gαh and the metastatic potential of TNBC, we performed immunoblotting to survey the endogenous level of Gαh in a panel of TNBC cell lines, including BT-20, BT549, HCC38, HCC1806, HCC1937, and MDA-MB-231. HCC1806 cells expressed the lowest Gαh level, whereas MDA-MB-231 cells displayed a relatively high level of Gαh expression (Fig. 2a). Accordingly, invasion assay revealed that HCC1806 cells exhibit a reduced invasive ability, and the MDA-MB-231 cells display an elevated invasive property among the tested TNBC cell lines (Fig. 2b). Statistical analysis showed that the endogenous protein levels of Gαh significantly (*p* = 0.042) correlates with cellular invasion ability in the tested TNBC cells (Fig. 2c). To understand if Gαh-OE is capable of promoting the invasiveness of TNBC cells, we ectopically expressed Gαh in HCC1806 cells (Fig. 2d). The data showed that Gαh-OE robustly increased the invasive ability of HCC1806 cells (Fig. 2e). Moreover, Gαh-OE appeared to significantly enhance the metastatic colony formation of HCC1806 cells in the lungs after tail vein injection into mice (Fig. 2f–h).

Conversely, we performed Gαh knockdown (KD) in MDA-MB-231 cells (Fig. 2i) to see if the transcriptional inhibition of Gαh could suppress the metastatic potential of TNBCs. Our data showed that silencing Gαh expression by two independent shRNA clones dramatically suppresses the invasive ability of MDA-MB-231 cells (Fig. 2j). Similarly, Gαh-KD appeared to effectively mitigate the metastatic colonization of MDA-MB-231 cells in the lungs of tumor-bearing mice (Fig. 2k–m). These findings demonstrate that Gαh-OE is likely correlated with metastatic progression of TNBCs.

### Intracellular GTP-binding activity, but not extracellular transglutaminase activity, of Gαh dictates the metastatic evolution of TNBCs

**Fig. 3:**
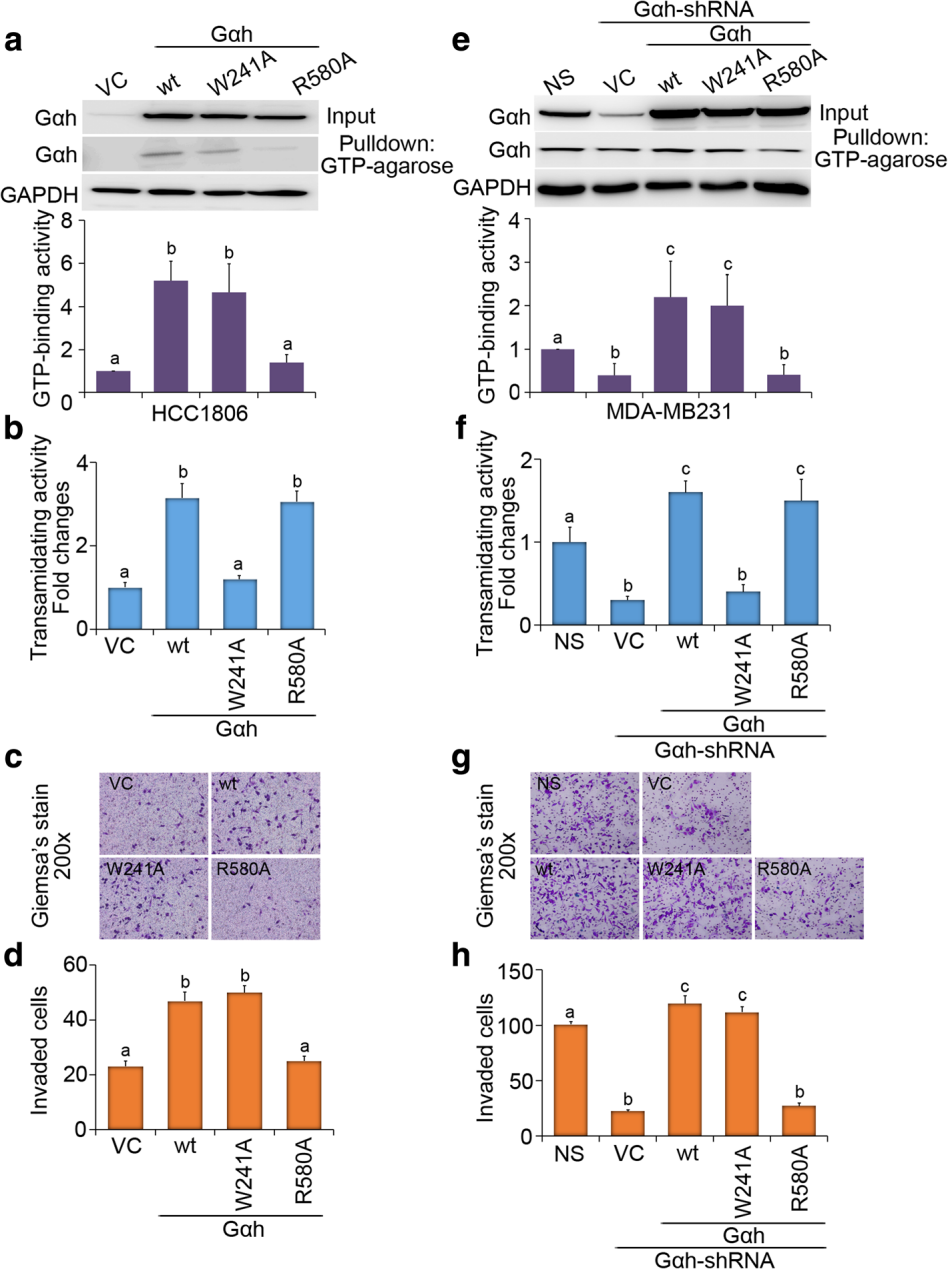
The GTP-binding, not transamidating, activity of Gαh determines the Gαh-induced invasiveness of TNBC cells. **a** Western blot analysis for Gαh, GTP-bound Gαh, and GAPDH expression in HCC1806 cells transfected with vector control (*VC*) or exogenous Gαh without (wild-type, *wt*) or with mutations at residues 241 (W to A) and 508 (R to A). The levels of GTP-bound Gαh from three independent experiments were normalized as fold changes relative to VC group and presented as mean ± SEM (*bottom*). **b** Transamidating activity assay of HCC1806 cell variants. **c** and **d** Giemsa staining **c** and quantification **d** of invaded cells after 16 h of incubation of HCC1806 cell variants. **e** Western blot analysis for Gαh, GTP-bound Gαh, and GAPDH expression in MDA-MB-231 cells transfected with non-silencing (NS) shRNA or Gαh-targeting shRNA followed by restoration of the expression of VC or exogenous wild-type and mutant Gαh. The levels of GTP-bound Gαh from three independent experiments were shown as fold changes relative to NS group and presented as mean ± SEM (*bottom*). **f** Transamidating activity assay of MDA-MB-231 cell variants. **g** and **h** **g** Giemsa staining and **h** quantification of invaded cells after 16 h of incubation of MDA-MB-231 cell variants. In **a**, **b**, **d**, **e**, **f**, and **h**, different *letters* above the columns indicate significant differences between the means (*p* < 0.05)

To delineate which Gαh activity is required for promoting the metastatic progression of TNBCs, we next performed site-directed mutagenesis to generate Gαh constructs with mutations at residues 241 (W to A) and 580 (R to A), which have been shown to result in defects in its transamidating and GTP-binding activities, respectively [17]. Our data showed that compared to wild-type Gαh, ectopic expression of the W241A mutant abolishes the transamidating, but not GTP-binding, activity of Gαh; conversely, the overexpression of R580A mutant inhibits the GTP-binding, but not transamidating, activity of Gαh in HCC1806 cells (Fig. 3a, b). Whereas the overexpression of the wild-type and W241A mutant Gαh appeared to enhance the invasive ability, ectopic expression of the R580A mutant failed to promote the invasive ability of HCC1806 cells (Fig. 3c, d).

To validate that the GTP-binding activity of Gαh is critical for mediating invasive activity of TNBCs, we next reconstituted the expression of wild-type and W241A/R580A mutant Gαh in Gαh-silenced MDA-MB-231 cells (Fig. 3e). Our results revealed that restoration of the expression of the wild-type and W241A mutant, but not the R580A mutant, Gαh rescues the GTP-binding activity and invasive ability of the Gαh-silenced MDA-MB-231 cells (Fig. 3e–h). Since restoring the expression of the transamidating activity-deficient W241A mutant was still incapable of recovering the invasive ability of Gαh-KD MDA-MB-231 cells, we concluded that the G protein function of Gαh dictates the metastatic evolution of Gαh-overexpressing TNBC cells.

### The upregulation of cytosolic, not extracellular, Gαh predicts poor prognosis and increased metastatic risk in breast cancer

**Fig. 4:**
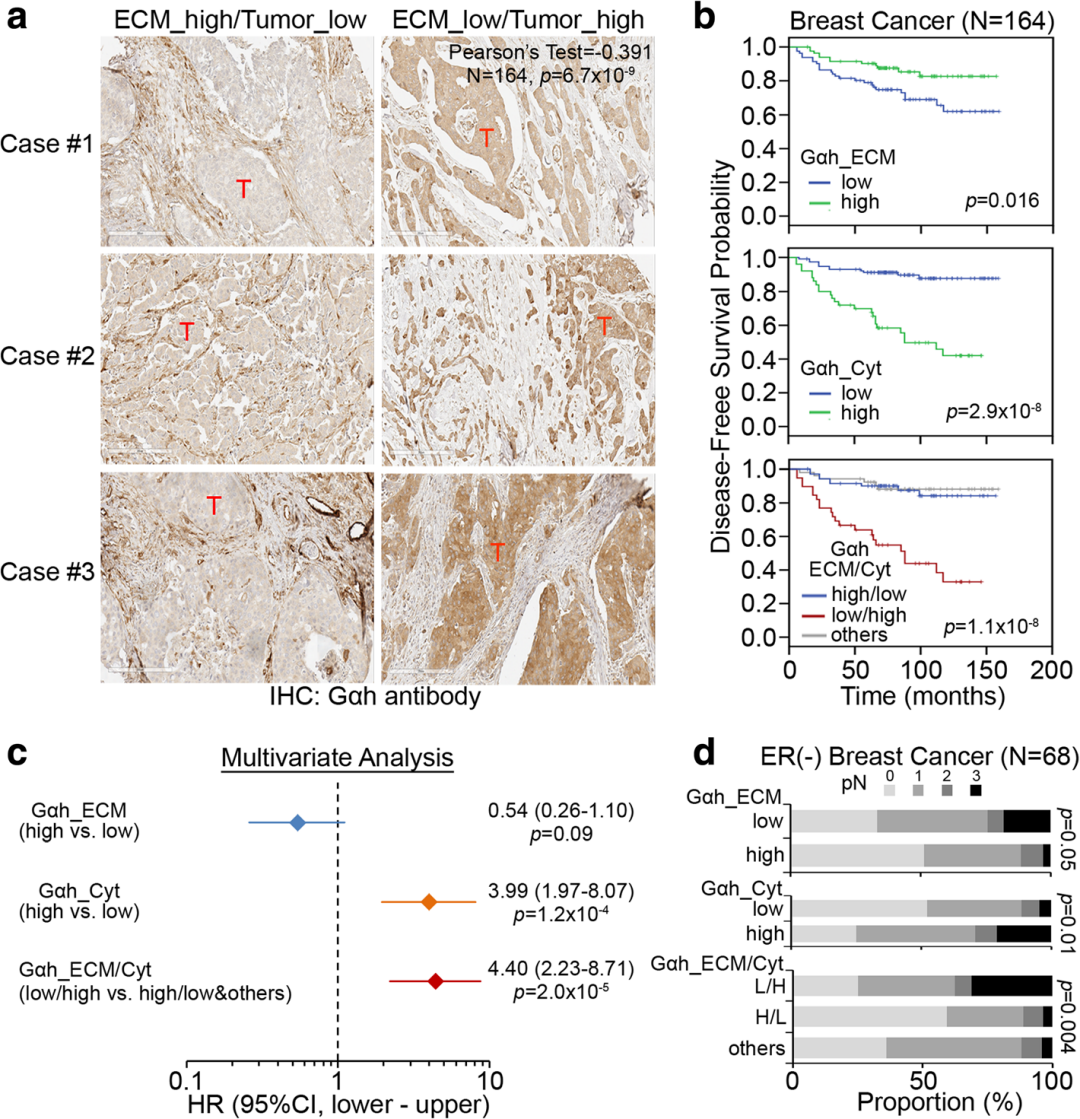
Clinical significance of extracellular and intracellular Gαh. **a** Representative images of IHC staining for Gαh in clinical breast cancer tissues. *T* denotes tumor regions. **b** Kaplan-Meier analysis for extracellular and intracellular Gαh levels determined by IHC staining in clinical breast cancer samples with the probability of disease-free survival (DFS). **c** Multivariate analysis adjusted by T and N stages for extracellular, intracellular or combined extracellular/intracellular Gαh levels using Cox regression test for the probability of DFS in breast cancer patients. **d** Correlation of extracellular, intracellular, or combined extracellular/intracellular Gαh levels with lymph node metastasis in ER-negative breast cancer

We next performed immunohistochemical (IHC) staining analysis to examine the protein level and distribution of Gαh in clinical specimens of breast cancers. Intriguingly, we found a pronounced accumulation of Gαh in either the extracellular matrix (ECM) or the cytosol in a subset of breast cancer tissues (Fig. 4a). Moreover, Gαh expression in the ECM and cytosol was significantly inversely correlated in the analyzed breast cancer tissues (Fig. 4a). Whereas increased levels of Gαh in the ECM predicted a favorable outcome, the accumulation of Gαh in the cytosol predicted a poor prognosis in breast cancer patients (Fig. 4b). In contrast to the signature of high extracellular and low cytosolic Gαh expression, low extracellular and high cytosolic Gαh levels appeared to be strongly correlated with a worse probability of disease-free survival in breast cancer patients (Fig. 4b). Cox regression analyses of disease-free survival demonstrated that increased levels of cytosolic Gαh, the signature of low extracellular/high cytosolic Gαh and higher T and N stages significantly correlate with an unfavorable hazard ratio, whereas the elevated levels of extracellular Gαh serves as a good prognostic factor in a univariate model (Additional file 1: Table S2). Notably, in the multivariate analysis adjusted by the prognostic power of higher T and N stages, high cytosolic Gαh and the low extracellular/high cytosolic Gαh signature remained as an independent risk factors for poor outcome among the studied breast cancer patients (Fig. 4c and Additional file 1: Table S3). Furthermore, in contrast to the high extracellular Gαh levels, our data revealed that the high cytosolic Gαh levels and the low extracellular/high cytosolic Gαh signature closely associates with lymph node metastasis in ER-negative breast cancer (Fig. 4d).

### The protein-protein interaction between Gαh and PLCδ1 determines cellular invasion in TNBC cells and serves as a therapeutic target for combating TNBC metastasis

**Fig. 5:**
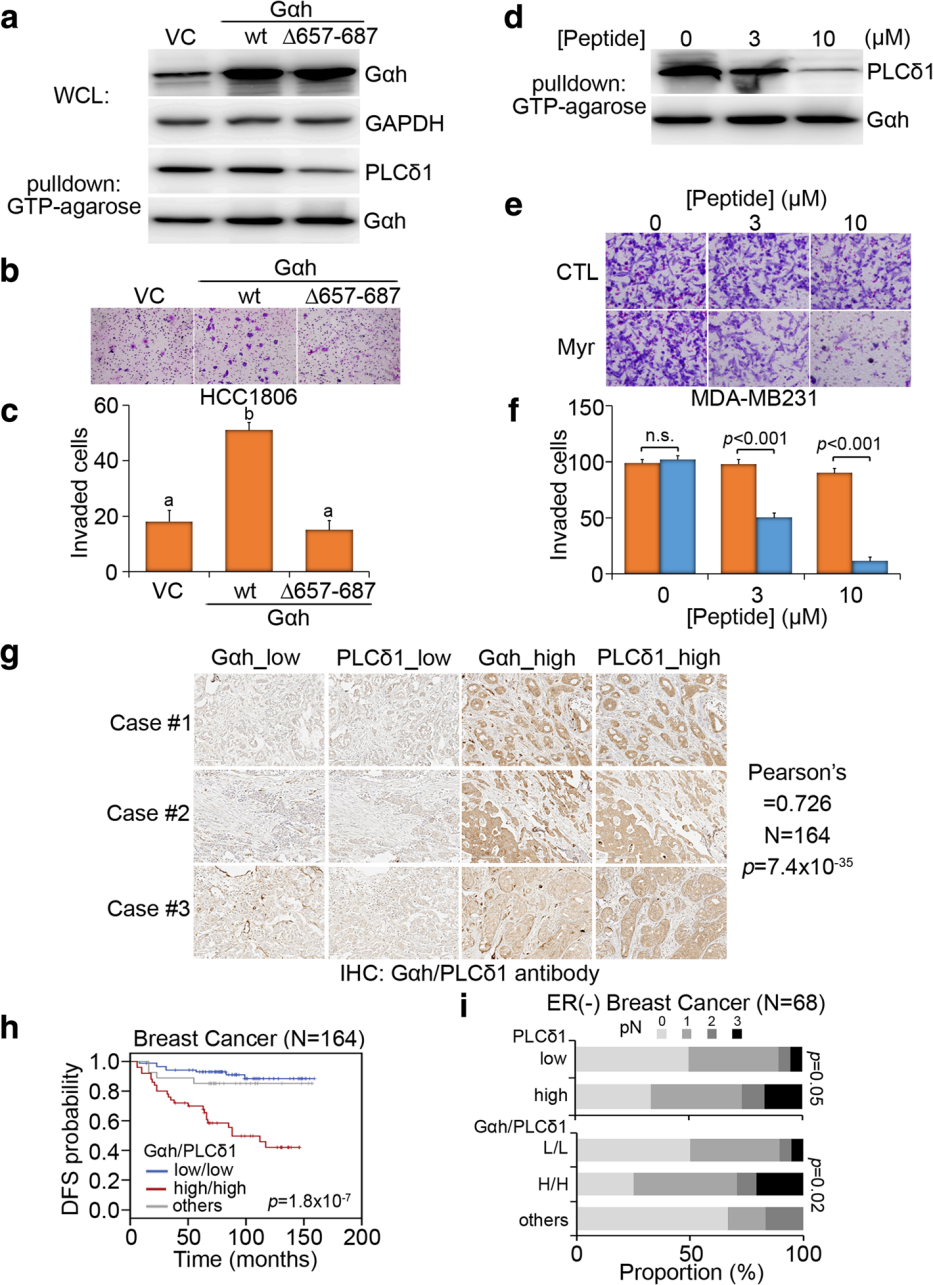
The protein-protein interaction between Gαh and PLC-δ1 regulates the invasive ability of TNBC cells and serves as a poor prognostic marker in breast cancer patients. **a** A schematic representation for the construction of the PLC-δ1-binding site deletion mutant of the Gαh protein. **b** and **c** Giemsa staining **b** and quantification (**c**) of the invaded H1806 cells after 16 h of transfection with empty vector (*VC*) or plasmid expressing wild-type (*wt*) or deletion-mutant (Δ657-687) Gαh. In **c**, different *letters* above the columns indicate significant differences between the means (*p* < 0.05). **d** Demonstration of the synthesis of the myristoylated (*Myr*) PLC-δ1 peptide. **e** and **f** **e** Giemsa staining and **f** quantification of invaded MDA-MB-231 cells treated with non-Myr (*control*, *CTL*, and *orange column*) or Myr-PLC-δ1 (*blue column*) peptide at the designated concentrations for 16 h. **g** IHC staining for Gαh and PLC-δ1 with their specific antibodies in breast cancer tissues. **h** Kaplan-Meier analysis of the Gαh/PLC-δ1 signature and the probability of DFS in breast cancer patients. **i** Correlation of PLC-δ1 or the Gαh/PLC-δ1 signature with lymph node metastasis in ER-negative breast cancer. In **c** and **f**, the data from three independent experiments were presented as the mean ± SEM

The coupling of activated Gαh to PLCδ1 signaling cascades has been identified previously [21]. To understand the importance of the protein-protein interaction (PPI) between Gαh and PLCδ1 in Gαh-promoted cellular invasion in TNBC cells, we next generated a Gαh variant (Δ657_687) with deletion of the PLCδ1-binding interface (between amino acids 657 and 677) (Fig. 5a). Notably, the ectopic expression of the Gαh/Δ657_687 mutant did not have any effect, whereas the wild-type Gαh increased the invasive ability of HCC1806 cells (Fig. 5b, c). We also synthesized a peptide fragment corresponding to the PLCδ1 amino acid sequence from 720 to 736, which has been identified previously as the binding-interface of Gαh [7], with or without myristoylation (Myr) at the N-terminus (Fig. 5d) in order to facilitate its penetration across the cell membrane. Cell invasion assay showed that the Myr-PLCδ1 peptide compared to the unmodified peptide suppressed the invasiveness of MDA-MB-231 cells in a dose-dependent manner (Fig. 5e, f). These findings suggest that the PPI between Gαh and PLCδ1 likely plays a central role in regulating the invasion of TNBC cells.

Moreover, IHC results from serial sections demonstrated that the levels of cytosolic Gαh and PLCδ1 are positively correlated (Fig. 5g). Similar to the high level of expression of cytosolic Gαh, increased protein levels of PLCδ1 was identified as poor prognostic factor in patients with breast cancer (Additional file 1: Figure S1A and Table S2) [22]. Moreover, the signature of the combination of high cytosolic Gαh and high PLCδ1 appeared to significantly predict a worse outcome in breast cancer patients (Fig. 5h). In addition, the Cox multivariate analysis of disease-free survival indicated that increased PLCδ1 level and the signature of high cytosolic Gαh/PLCδ1 were independent predictive factors of poor outcomes in breast cancer patients (Additional file 1: Figure S1B and Table S4). Notably, the high level of PLCδ1 and the high cytosolic Gαh/PLCδ1 signature correlated with higher pathologic and N stage in ER-negative breast cancer patients (Fig. 5i). These results highlight the therapeutic value of targeting the PPI of the Gαh/PLCδ1 complex in combating metastasis of TNBC.

**Fig. 6:**
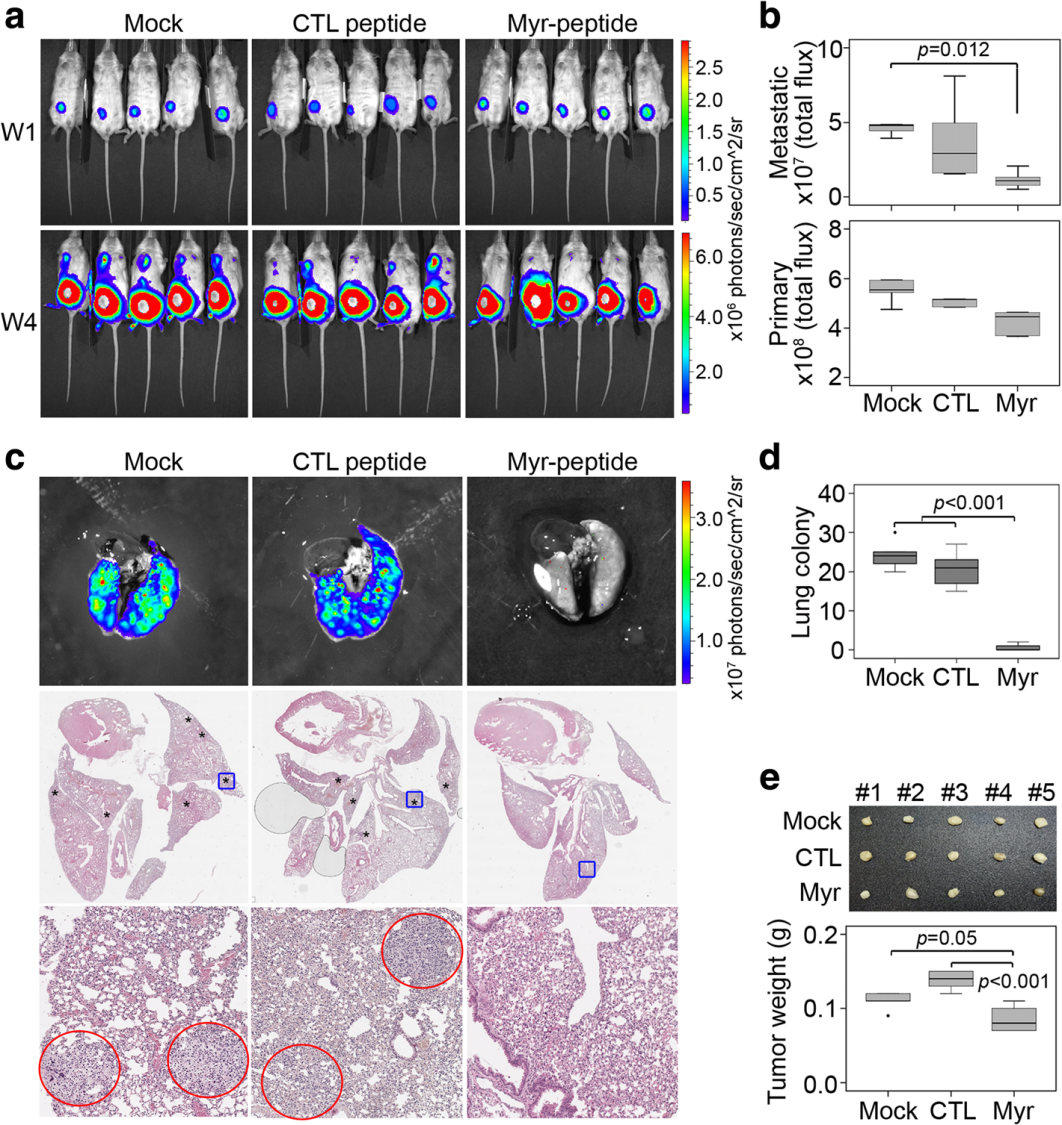
Therapeutic effectiveness of targeting the PPI of Gαh/PLC-δ1 by Myr-PLC-δ1 peptide in the orthotropic mouse model of breast cancer. **a** and **b** The luminescence intensity of luciferase-expressing MDA-MB-231 cells on weeks 1 and 4 after the orthotropic transplantation into mammary fat pads mice (**a**). Mice (*n* = 5) were treated without [PBS containing 0.1% TFA (Mock)] or with CTL or Myr-PLC-δ1 peptide on week 1. **b** Luminescence intensities of the total flux of protons in the metastatic sites and primary tumors in each group were presented as the median ± SD. **c** The representative luminescent images (*upper panel*) and IHC staining images (*middle* and *lower panels*) of lungs from each group at the endpoint. **d** Quantitation of the lung colonies in **c. e** Size and weight of primary tumors obtained from each group on week 4. The tumor weights are presented as the median ± SD. In **b**, **d**, and **e**, significant differences were analyzed by one-way ANOVA using Tukey’s test

To validate the therapeutic utility of disrupting the PPI between Gαh and PLCδ1 to suppress TNBC metastasis, we next generated an orthotropic mouse model with luciferase-expressing MDA-MB-231 cells (Fig. 6a). Our data showed that administration of Myr-PLCδ1 compared to that of the control peptide robustly inhibited distant lung metastasis (Fig. 6a, b) accompanied with a reduced tumor growth (Fig. 6b) of MDA-MB-231 cells at the primary site in tumor-bearing mice 3 weeks post-treatment. Moreover, histological analysis revealed that the formation of tumor colonies in the lungs was clearly suppressed in the orthotropic mouse model of breast cancer after the treatment with Myr-PLCδ1 peptide compared to that with the control peptide for 3 weeks (Fig. 6c, d). Significantly, in comparison with the control groups, the administration of Myr-PLCδ1 peptide appeared to reduce tumor weight at the end of treatment (Fig. 6e). Therefore, the PPI of the Gαh/PLCδ1 complex is not only critical for relaying TNBC metastasis but also serves as a useful target for developing anti-cancer agents against metastatic TNBCs.

## Discussion

The oncogenic role of extracellular and cytosolic Gαh in promoting cancer progression is still controversial. The increased levels of extracellular Gαh have been shown to promote breast cancer metastasis by recruiting integrin-related signaling cascades [10, 23]. Similar findings were found in ovarian cancer where extracellular Gαh promotes metastasis via activating the NF-κB signaling axis [24]. Conversely, here, we show that the increased levels of extracellular Gαh refers to a favorable prognosis in breast cancer patients. Similarly, an increased level of extracellular Gαh was previously shown to inhibit tumor invasion in TNBCs [25]. Moreover, our data showed that the loss of the transamidating, but not G protein, function of Gαh is capable of rescuing the reduced invasive ability in Gαh-silenced MDA-MB-231 cells. Similarly, it has been demonstrated that the catalytically inactive (C277S) mutant of Gαh activates NF-κB and induces HIF-1α expression as effectively as wild-type Gαh [26]. Since secreted Gαh has been thought to be responsible for catalyzing the crosslinking of ECM proteins via its transamidating activity [10], our findings suggest that extracellular Gαh likely functions as a tumor suppressor in TNBCs.

Here, we show that the pronounced accumulation of Gαh in the cytoplasm predicts worse prognosis and causally associates with the expression of PLC-δ1 in breast cancer. The loss of both the G protein function of Gαh and its PLC-δ1 interacting domain reduces the Gαh-enhanced metastatic potential of TNBC cells. Previous studies have shown that activation of the Gαh/PLC-δ1 signaling axis by several G protein-coupled receptors (e.g., α1B-adrenergic [5], oxytocin [27], and FSH [6] receptors) induces fluctuations in the levels of intracellular Ca^2+^ and promotes the metastatic evolution of TNBC cells by modulating their epithelial-to-mesenchymal transition (EMT), which is known as the initial step of cancer metastasis [28, 29]. Notably, the activation of FSH receptor has been shown to induce EMT by triggering the PI3K/Akt-Snail signaling pathway in ovarian cancer cells [30] and to enhance cellular migration and invasion by modulating actin cytoskeleton activity in breast cancer cells [31]. Accordingly, the activation of oxytocin receptor has been found to elevate the invasive properties of endometrial cancer cells via the PI3K/Akt axis [32] and to promote the migratory ability of prostate cancer cells by coupling to the Gi-dependent pathway [33]. These findings implicate the possible role of the Gαh/PLC-δ1 signaling axis in these events since the activation of the Gαh/PLC-δ1 pathway has been identified to elicit intracellular Ca^2+^ elevation, promote IP3 turnover, and modulate myosin and actin dynamics [34].

Several studies have demonstrated that Gαh-OE correlates with metastatic progression [35–39] as well as drug resistance [40, 41] in different types of cancer, suggesting that the discovery of inhibitors of PPI in the Gαh/PLC-δ1 complex could be a novel and useful strategy to combat malignant tumors. Although therapeutic targeting of the PPI of Gαh/PLC-δ1 is likely to be effective in treating metastatic TNBCs, further experiments are still needed to identify the Gαh-coupled receptor(s) and related signaling cascades in TNBC cells.

## Conclusions

Our data delineated the inverse prognostic values of extracellular and cytoplasmic Gαh and validated that the G-protein activity of Gαh is crucial for promoting TNBC metastasis by recruiting the PLC-δ1-associated signaling axis. Blocking the PPI between Gαh and PLC-δ1 displayed an inhibitory effect on the metastatic capacity of TNBC cells in vitro and in vivo, providing a new avenue for drug discovery in cancer.

## Additional file

Additional file 1: Table S1. The paired primers used in the study. **Table S2.** Cox univariate analysis of disease-free survival for the protein of cytosol Gαh [Gαh (C)], extracellular Gαh [Gαh (E)] and PLC-δ1 and the stage of pathologic T and N. **Table S3.** Cox multivariate analysis of disease-free survival for the protein of Gαh (C) and Gαh (E) and the stage of pathologic T and N. **Table S4.** Cox multivariate analysis of disease-free survival for the protein of Gαh (C) and PLC-δ1 and the stage of pathologic T and N. **Figure S1.** Clinical relevance of Gαh and PLC-δ1 in breast cancer patients. (DOCX 223 kb)
