# Supplementary material for: The Gαh-PLCδ1 signaling axis drives metastatic progression in triple-negative breast cancer
Source: J Hematol Oncol. 2017 Jun 2;10:114. doi: 10.1186/s13045-017-0481-4 (PMC5457652; doi:10.1186/s13045-017-0481-4)
Supplement: Additional file 1: Table S1. — The paired primers used in the study. Table S2. Cox univariate analysis of disease-free survival for the protein of cytosol Gαh [Gαh (C)], extracellular Gαh [Gαh (E)] and PLC-δ1 and the stage of pathologic T and N. Table S3. Cox multivariate analysis of disease-free survival for the protein of Gαh (C) and Gαh (E) and the stage of pathologic T and N. Table S4. Cox multivariate analysis of disease-free survival for the protein of Gαh (C) and PLC-δ1 and the stage of pathologic T and N. Figure S1. Clinical relevance of Gαh and PLC-δ1 in breast cancer patients. (DOCX 223 kb) [file 13045_2017_481_MOESM1_ESM.docx]

**Supplementary Information**

**The Gαh-PLCδ1 signaling axis drives metastatic progression in triple-negative breast cancer**

Shang-Pen Huang, Pei-Yao Liu, Chih-Jung Kuo, Chi-Long Chen, Wei-Jiunn Lee, Yu-Hui Tsai and Yuan-Feng Lin

**Table of contents**

**Table S1.** The paired primers used in the study.

**Table S2.** Cox univariate analysis of disease-free survival for the protein of cytosol Gαh [Gαh (C)], extracellular Gαh [Gαh (E)] and PLC-δ1 and the stage of pathologic T and N.

**Table S3.** Cox multivariate analysis of disease-free survival for the protein of Gαh (C) and Gαh (E) and the stage of pathologic T and N.

**Table S4.** Cox multivariate analysis of disease-free survival for the protein of Gαh (C) and PLC-δ1 and the stage of pathologic T and N.

**Figure S1.** Clinical relevance of Gαh and PLC-δ1 in breast cancer patients.

**Table S1.** The paired primers used in the study.

| **Gαh:**   \| Forward primer (IRES2_EGFP): A-TCGAGATGGCCGAGGAGCTGGTCTTAG \| \| --- \| \| B-GATGGCCGAGGAGCTGGTCTTAG \| \| Forward primer (pLAS3W): A-CTAGCATGGCCGAGGAGCTGGTCTTAG  B-CATGGCCGAGGAGCTGGTCTTAG  Reverse primer: C-AATTCTTAGGCGGGGCCAATGATGAC \| \| D-CTTAGGCGGGGCCAATGATGAC  **W241A:**  Forward primer (flanking): CTAGCTAGCATGGCCGAGGAGCTGGTC  Reverse primer (flanking): CGGAATTCTTAGGCGGGGCCAATGATGA  Forward primer (internal): GGACGCGCGGACAACAACTA  Reverse primer (internal): TAGTTGTTGTCCGCGCGTCC  **R580A:**  Forward primer: ACCTGCTGGCTGAGGCGGACCTCTACCTGG \|   Reverse primer: CCAGGTAGAGGTCCGCCTCAGCCAGCAGGT  **Δ657-687:**  Forward primer: CTAGCTAGCATGGCCGAGGAGCTGGTC  Reverse primer: CGGAATTCTTACGGCAGCAGGTCCATTG |
| --- | --- | --- | --- | --- |

*The recognition sequence of restriction enzyme was underlined.

**Table S2.** Cox univariate analysis of disease-free survival for the protein of cytosol Gαh [Gαh (C)], extracellular Gαh [Gαh (E)] and PLC-δ1 and the stage of pathologic T and N.

|  | **HR** | **95.0% CI for HR** | | ***P*** |
| --- | --- | --- | --- | --- |
| **Variables** |  | **Lower** | **Upper** |  |
| Gαh (E) high vs. low | 0.442 | 0.222 | 0.880 | 0.02 |
| Gαh (C) high vs. low | 5.614 | 2.817 | 11.189 | <0.0001 |
| Gαh E_low/C_high vs. others | 5.957 | 3.084 | 11.505 | <0.0001 |
| PLC-δ1 high vs. low | 4.684 | 2.140 | 10.249 | <0.0001 |
| TGM2 C_high/PLCD1_high vs. others | 5.614 | 2.817 | 11.189 | <0.0001 |
| T34 vs. T12 | 2.802 | 1.439 | 5.457 | 0.002 |
| N123 vs. N0 | 7.285 | 2.823 | 18.801 | <0.0001 |

**Table S3.** Cox multivariate analysis of disease-free survival for the protein of Gαh (C) and Gαh (E) and the stage of pathologic T and N.

|  | **HR** | **95.0% CI for HR** | | ***P*** |
| --- | --- | --- | --- | --- |
| **Variables** |  | **Lower** | **Upper** |  |
| Gαh (E) high vs. low | 0.539 | 0.263 | 1.102 | 0.090 |
| T34 vs. T12 | 1.320 | 0.645 | 2.702 | 0.448 |
| N0_N123 | 5.743 | 2.120 | 15.561 | 0.001 |
| Gαh (C) high vs. low | 3.990 | 1.972 | 8.072 | <0.0001 |
| T34 vs. T12 | 1.400 | 0.694 | 2.821 | 0.347 |
| N123 vs. N0 | 4.828 | 1.785 | 13.057 | 0.002 |
| Gαh C_high/E_low vs. others | 4.403 | 2.227 | 8.705 | <0.0001 |
| T34 vs. T12 | 1.291 | 0.632 | 2.640 | 0.483 |
| N123 vs. N0 | 4.723 | 1.724 | 12.939 | 0.003 |

**Table S4.** Cox multivariate analysis of disease-free survival for the protein of Gαh (C) and PLC-δ1 and the stage of pathologic T and N.

|  | **HR** | **95.0% CI for HR** | | ***P*** |
| --- | --- | --- | --- | --- |
| **Variables** |  | **Lower** | **Upper** |  |
| PLC-δ1 high vs. low | 3.529 | 1.595 | 7.808 | 0.002 |
| T34 vs. T12 | 1.466 | 0.728 | 2.955 | 0.284 |
| N123 vs. N0 | 4.692 | 1.728 | 12.744 | 0.002 |
| Gαh C_high/PLC-δ1_high vs. others | 3.990 | 1.972 | 8.072 | <0.0001 |
| T34 vs. T12 | 1.400 | 0.694 | 2.821 | 0.347 |
| N123 vs. N0 | 4.828 | 1.785 | 13.057 | 0.002 |


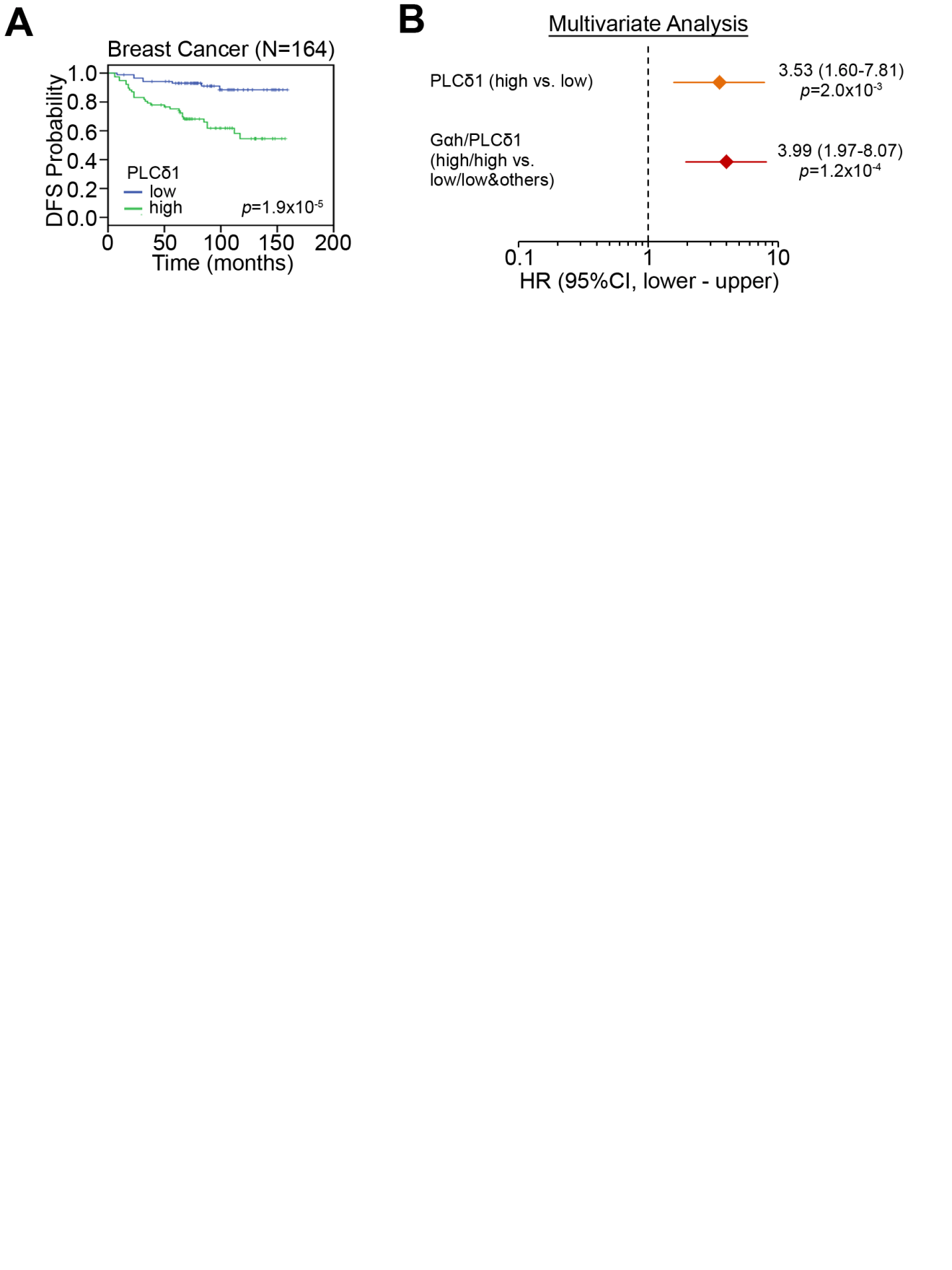


**Figure S1.** Clinical relevance of Gαh and PLC-δ1 in breast cancer patients. (A) Kaplan-Meier analysis for PLC-δ1 levels in clinical breast cancer patients under DFS probability. (B) Multivariate analysis adjusted by T and N stage for PLC-δ1 or Gαh/PLC-δ1 signature levels using Cox regression test under the DFS probability against clinical breast cancer patients.
